# WE CAN WORK IT OUT: NURSING HOME SOCIAL WORK AND CONFLICT MEDIATION

**DOI:** 10.1093/geroni/igad104.0106

**Published:** 2023-12-21

**Authors:** Amy Roberts, Nancy Kusmaul, Mercedes Bern-Klug

**Affiliations:** Miami University, Oxford, Ohio, United States; University of Maryland Baltimore County, Baltimore, Maryland, United States; University of Iowa, Iowa City, Iowa, United States

## Abstract

Drawing from a nationally representative survey of social service directors (N=922), this presentation will provide an overview of nursing home staffing trends and describe the ways in which social service staff routinely interact with residents, families, and staff. Social workers are involved in many aspects of care, including the admissions process, planning and providing care, care transitions, and general supportive services. As members of the interdisciplinary team, social workers advocate for resident rights, and support decision-making and care planning in times of family crisis and high conflict situations. Summary statistics will be presented to identify the directors’ frustrations with the role – including their perceptions of major barriers to care, and draw attention to the changes that would improve job satisfaction (e.g., higher salary/wage, 51%; lower staff turnover among direct care staff, 47%; and more time to focus on the social and emotional concerns of residents, 45%). The presentation will conclude with an overview of directors’ interest in training topics that may help to mediate conflicts among residents, families, and staff. Specifically, we will share how prepared directors feel to train other staff in conflict-resolution skills (e.g., working with the resident/family and team to balance resident self-determination with the nursing home’s responsibility to minimize risk), and the use of an ethical framework to guide action.

